# Updated diagnosis and graft involvement for visceral leishmaniasis in kidney transplant recipients: a case report and literature review

**DOI:** 10.1007/s15010-022-01943-3

**Published:** 2022-11-03

**Authors:** Marco Busutti, Alessandro Deni, Alessandra Mistral De Pascali, Margherita Ortalli, Luciano Attard, Bianca Granozzi, Benedetta Fabbrizio, Gaetano La Manna, Giorgia Comai, Stefania Varani

**Affiliations:** 1 Nephrology, Dialysis and Renal Transplant Unit, IRCCS-Azienda Ospedaliero-Universitaria di Bologna, Bologna, Italy; 2 Department of Experimental, Diagnostic and Specialty Medicine, Alma Mater Studiorum University of Bologna, Bologna, Italy; 3 Infectious Diseases Unit, IRCCS Azienda Ospedaliero-Universitaria di Bologna, Bologna, Italy; 4 Pathology Unit, IRCCS, Azienda Ospedaliero-Universitaria di Bologna, Bologna, Italy; 5 Unit of Clinical Microbiology, IRCCS Azienda Ospedaliero-Universitaria di Bologna, Bologna, Italy

**Keywords:** Leishmania, Transplantation, Kidney, Infection

## Abstract

**Purpose:**

Visceral leishmaniasis (VL) has become a rising concern to transplantation teams, being associated with graft dysfunction and reduced survival of renal transplant recipients. Here, we describe a case of VL occurring in a kidney transplant (KT) recipient in Italy, a country in which *Leishmania infantum* is endemic and we reviewed the literature on the clinical course and diagnosis of VL in KT recipients residing or travelling to southern Europe.

**Results:**

The VL case was diagnosed 18 months after transplant and 28 days after the onset of symptoms by quantitative PCR (qPCR) on peripheral blood. A graft biopsy showed renal involvement, and PCR performed on graft tissue displayed the presence of *Leishmania* DNA. The retrospective confirmation of *Leishmania*-positive serology in a serum sample collected before transplantation, as well as the absence of anti-*Leishmania* IgG in the graft donor strongly suggest that reactivation of a latent parasitic infection caused VL in the current case.

**Conclusion:**

VL is often underdiagnosed in transplant recipients, despite the presence of latent *Leishmania* infection being reported in endemic countries. This case report, as well as the literature review on leishmaniasis in KT recipients, underline the importance of rapid VL diagnosis to promptly undergo treatment. Serology is scarcely sensitive in immunocompromised patients, thus molecular tests in peripheral blood should be implemented and standardized for both VL identification and follow-up.

## Introduction

Visceral leishmaniasis (VL) is an infectious disease caused by protozoa of the *Leishmania donovani* complex*,* and associated with considerable morbidity and mortality [1]. VL is also a known complication of solid organ transplantation (SOT) [2]; the prevalence of VL among SOT recipients in endemic areas is up to 0.9% [3, 4]. In these patients, VL can occur either *ex novo* or through reactivation of a pre-existing infection induced by the immunosuppressive drugs. In kidney transplant (KT) recipients, leishmaniasis often includes acute interstitial nephritis with moderate inflammation and infiltration of lymphocytes, plasma cells and macrophages, and can lead to graft dysfunction [5]. VL occurring in SOT is a severe event, being associated with frequent relapses and a mortality rate that exceeds 20% [6].

Here, we report a VL case in a KT recipient residing in central Italy, where *L. infantum* circulates. We diagnosed and monitored VL by quantitative PCR (qPCR) and retrospectively screened pre-transplant samples of the index patient and the graft donor by serology. We also briefly reviewed the literature on the clinical course and diagnosis of VL in KT recipients residing or travelling to southern Europe.

## Case presentation

A 36-year-old Caucasian male underwent pre-emptive kidney transplantation at the Nephrology, Dialysis and Renal Transplant Unit, University Hospital of Bologna, Italy, for end-stage renal disease (ESRD) secondary to autosomal dominant polycystic kidney disease (ADPKD). The graft was available from a living donor (a first-degree relative). Induction therapy with high dose steroids and anti-IL2r monoclonal antibodies (basiliximab) was first administered after transplant, followed by maintenance therapy with prednisone, tacrolimus and mycophenolic acid. No major complications were reported, and graft function was stabilized with serum creatinine (sCreat) levels at 2 mg/dl (eGFR CKD-EPI 42 ml/min).

Approximately 18 months after transplant, the patient was admitted following a routine follow-up appointment during which he complained of night sweat and persistent mild fever in the previous 3 weeks. Symptoms had been empirically treated with oral antibiotics by the patient’s general practitioner, with no benefit. Laboratory examination showed pancytopenia (WBC 2370/mmc, Hb 10.5 g/dl, PLT 76.000/mmc), renal impairment (sCreat 3.7 mg/dl, eGFR CKD-EPI 19 ml/min), elevated C-reactive protein (CRP 12 mg/dl), and elevated ferritin (902 ng/ml). 18F-FDG PET/C was performed, showing splenomegaly and splenic hypercaptation. Since a discrepancy between the donor’s and the recipient’s serostatuses for human cytomegalovirus (CMV) was known (D + /R −), a primary CMV infection was initially suspected, and mycophenolic acid was suspended at admission. As CMV DNAemia tested negative, the suspicion of CMV disease was dismissed.

**Fig. 1:**
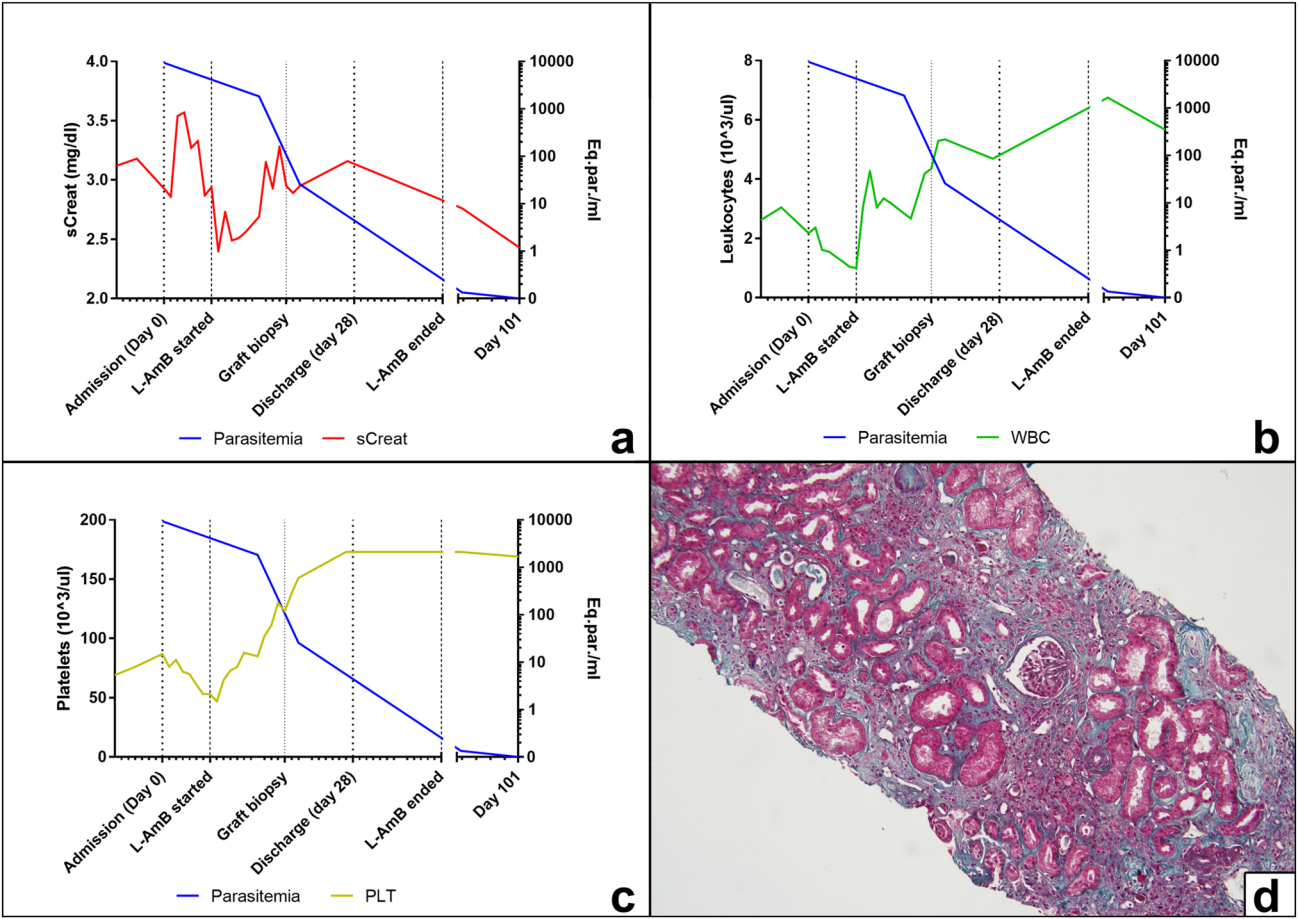
Molecular diagnosis and monitoring and histological evaluation of visceral leishmaniasis in a kidney transplant recipient. **a**–**c** Time course of parasitological and laboratory parameters in the index patient. Parasitaemia was measured by quantitative real-time PCR (qPCR) before and after anti-leishmanial treatment (L-Amb). Detection of kDNA by qPCR was set up as described in Mary et al. [8]. The standard curve was created from *Leishmania* DNA extracted from 5 × 10^6^ promastigotes of the *L. infantum* reference strain MHOM/TN/80/IPT1, performing serial dilution to 0.0005 parasites. Detection of kDNA reached the sensitivity of 0.05 parasite equivalents/ml. d. Histology examination of the kidney biopsy shows interstitial fibrosis and tubular atrophy (Trichrome stain, 10×). sCreat; serum creatinine levels. WBC; white blood cells. *PLT* platelets. *Eq.par.* equivalent parasites

Two real-time (rt)PCR assays targeting the small-subunit ribosomal (r)RNA gene and the leishmanial kinetoplast (k)DNA, respectively, were simultaneously performed on peripheral whole blood as described by Varani et al. [7]. Quantification of parasitic kinetoplast (k)DNA was also performed. At diagnosis, the parasite load was 9360 parasite equivalents/ml (Fig. 1a–c). VL serology was carried out by rK39-based immunochromatographic test (ICT; Rapydtest, Diagnostic International Distribution S.p.A, Milan, Italy), by enzyme-linked immunosorbent assay (ELISA, Vircell, Granada, Spain) and by immunofluorescence assay (IFA, BioMérieux, Marcy-l’Étoile, France). The sample tested positive on ELISA and negative on the ICT, while the IFA returned a title of 1:80, corresponding to an indeterminate result.

As the renal function worsened, a graft biopsy was performed. Histological examination of the biopsy showed diffuse interstitial fibrosis/tubular atrophy with moderate chronic interstitial inflammation and glomerulosclerosis in almost 50% of glomeruli associated to chronic vascular damage (Fig. 1d). CD1a staining was negative and parasite amastigotes were not detected at histology nor at electron microscopy, while parasitic kDNA was identified by rtPCR in renal tissue (data not shown). Bone marrow biopsy was also carried out, showing myelodysplasia, which was consistent with VL. Unfortunately, this sample was not sent to the Microbiology Unit and kDNA rtPCR was not performed.

Anti-leishmanial therapy with liposomal amphotericin B (L-Amb) was carried out (eight infusions; 5 mg/kg/dose) with good response. During treatment, both thrombocytopenia and leukopenia rapidly improved, and a decrease of sCreat levels was also observed after a brief initial increase, likely due to L-AmB-related nephrotoxicity. rtPCR for *Leishmania* tested negative on peripheral blood within 3 months after the first L-AmB infusion (Fig. 1a–c). Graft function partially recovered, with sCreat stabilizing at 2.1–2.5 mg/dl at 15 months after VL diagnosis and further decreasing to 1.8–2.0 mg/dl at 42 months; at this time the patient was also free from VL relapses, with no *Leishmania* DNA detected in peripheral blood samples. Administration of mycophenolic acid was not reinstated after treatment.

The serostatus for *Leishmania* in the index patient and the graft donor was also retrospectively analysed by testing sera that were collected immediately before the transplant with the *Leishmania* Western Blot IgG (LDBio Diagnostics^®^, Lyon, France), a sensitive method to detect specific IgG in individuals with VL or asymptomatic *Leishmani*a infection [9, 10]. The test revealed the presence of anti-leishmanial IgG in the patients’ serum immediately before the transplant, while the donor’s serum tested negative.

## Methods

We conducted a review of case reports and case series published in the MEDLINE (PubMed) database between 1990 and 2021 using the following search terms: *“Leishmania*”, “transplant” and their derivatives, and excluding results relative to non-European countries, cutaneous leishmaniasis and haematological transplants. Furthermore, the bibliographies of extant case series were searched for relevant articles. The results included articles in English, French and Spanish.

Data were entered into a pre-designed Excel file. Information extracted from each paper was (1) country where the infection likely took place; (2) characteristics of patients (sex, age); (3) immunosuppressive treatment; (4) time to VL after transplant; (4) clinical and laboratory markers (5) histology of renal biopsy; (6) methods of VL diagnosis; (7) VL treatment; (8) outcome after treatment (including recovery, relapse, death).

## Discussion

### Epidemiology and transmission

VL is an underrecognized and misdiagnosed disease among transplant recipients [6]. According to our review of the extant literature (Table 1), 61 VL cases (including the current case) have been reported in KT recipients in southern Europe, or in patients who travelled there, since 1990. Most cases (*n = *23) were reported in Spain, which is among the foremost countries in performing organ transplantation and in which *L. infantum* is endemic [11], followed by France (*n = *15), Italy (*n = *12, including the case of this study) and Greece (*n = *5); Turkey reported two cases, and Portugal and Malta reported one VL case each. Finally, two cases were described in patients residing in non-endemic countries, but reporting travelling to Spain. Thus, VL is a threat for SOT recipients in areas where leishmaniasis is endemic.

**Table 1:** Cases of human VL in SOT recipients reported in literature between 1990 and 2020

Year	Author	No. of cases	Country	Immuno-suppressive treatment	Time to VL after Tx	Clinical markers	Laboratory markers	Histology of renal biopsy	Method(s) of VL diagnosis	Treatment of VL	Relapses/outcome
1990	Donovan et al. [12]	1	Malta	AZA + PSL	78 months	Lethargy; anorexia; weight loss	Pancytopenia	NR	BM microscopy	SSG	Relapse
1990	Le Cacheux et al. [13]	1	France	AZA + PS	168 months	Fever; splenomegaly; hepatomegaly; fatigue	Pancytopenia	NR	BM microscopy; serology (IFA)	MA	NR
1991	Rousaud et al. [14]	1	Spain	AZA + PS	25 months	Fever; splenomegaly	Pancytopenia; hypergammaglobulinemia; hypoalbuminemia	NR	BM microscopy; serology (NR)	MA	Relapse
1992	Orofino et al. [15]	2	Spain	AZA + PS (*n = *1); CsA + PS (*n = *1)	5 months (*n = *2)	Fever (*n = *2); splenomegaly (*n = *2); hepatomegaly (*n = *1); malaise (*n = *1); fatigue (*n = *1)	Leukopenia (*n = *2); thrombocytopenia (*n = *1)	NR	BM microscopy (*n = *2)	MA (*n = *2)	Relapse (*n = *1)
1992	Moulin et al. [16]	1	France	Cs + PS	5 months	Fever; chills; splenomegaly	Leukopenia; thrombocytopenia; hypergammaglobulinemia	NR	BM microscopy; serology (IFA)	MA + allopurinol	NR
1992	Jokipii et al. [17]	1	Finland (travel to Spain)	AZA + MPSL + Cs	4 months	Fever; non-productive cough	Anaemia; leukopenia; hypergammaglobulinemia	Fine needle aspiration revealed amastigotes in renal monocytes	Bronchoalveolar lavage microscopy; BM microscopy and culture; serology (IFA)	SSG	NR
1993	Dunan et al. [18]	4	France	NR	5 months (*n = *1); 6 months (*n = *1); 51 months (*n = *1); 168 months (*n = *1)	Fever (*n = *3); splenomegaly (*n = *1); NR (*n = *1)	Pancytopenia (*n = *1); anaemia (*n = *1); NR (*n = *2)	NR	BM microscopy (*n = *4); serology (WB) (*n = *4)	MA (*n = *3); AmB (*n = *1)	Relapse (*n = *1); died during treatment (*n = *1)
1993	Torregrosa et al. [19]	1	Spain	Cs + AZA + PS	8 months	Fever; splenomegaly	Pancytopenia	NR	BM microscopy	MA	NR
1995	Moroni et al. [20]	1	Italy	PS + AZA	15 months	Fever; malaise; weight loss; non-productive cough; hepatomegaly; splenomegaly	Pancytopenia; hypergammaglobulinemia; elevated liver enzymes	NR	BM microscopy; BM culture; serology (ELISA)	MA + allopurinol	Died from pneumonia during treatment
1996	Esteban et al. [21]	1	Spain	Cs + AZA + PS	12 months	Fever; abdominal pain	Pancytopenia; elevated sCreat	NR	BM microscopy	MA	NR
1996	Torrús et al. [22]	1	Spain	NR	NR	Fever	Pancytopenia	NR	BM microscopy; BM culture	MA (discontinued due to intolerance), ketoconazole + allopurinol	Relapse
1997	Apaydin et al. [23]	1	Turkey	PSL + AZA	52 months	Splenomegaly; weight loss	Anaemia; leukopenia; hypergammaglobulinemia; elevated liver enzymes; elevated CRP	NR	Serology (ELISA); BM microscopy	MA	Relapse
1998	Berenguer et al. [24]	1	Spain	Cs + AZA + PS	40–48 months	Fever; hepatomegaly; splenomegaly	Pancytopenia	NR	BM microscopy and culture; serology (IFA)	MA then L-AmB	NR
1998	Gomez-Campedera et al. [25]	1	Spain	NR	48 months	Fever; hepatomegaly; splenomegaly	Pancytopenia	NR	BM microscopy; BM culture; serology (IFA)	MA (discontinued due to intolerance), L-AmB	NR
1999	Boletis et al. [26]	4	Greece	Cs + MPSL + AZA (*n = *4)	5 months (*n = *1); 8 months (*n = *1); 19 months (*n = *1); 54 months (*n = *1)	Fever (*n = *4); splenomegaly (*n = *3)	Pancytopenia (*n = *2); NR (*n = *2)	NR	BM microscopy (*n = *3); serology (IFA) (*n = *4); BM PCR (*n = *1)	L-AmB (*n = *3); MA (*n = *1)	Graft loss immediately before symptoms onset (*n = *1)
1999	Hernandez-Perez et al. [27]	5	Spain	AZA + PS (*n = *3); Cs + AZA + PS (*n = *2)	6 months (*n = *1); 18 months (*n = *1); 115 months (*n = *1); NR (*n = *2)	Fever (*n = *5); splenomegaly (*n = *4)	Pancytopenia (*n = *5); disseminated intravasal coagulation (*n = *1)	Necropsy revealed presence of *Leishmania* amastigotes in the kidney	BM microscopy (*n = *3); serology (NR) (*n = *3); necroscopy (*n = *1)	MA (*n = *4); untreated (*n = *1)	Relapse (*n = *2); died shortly after VL onset (*n = *3)
1999	Hueso et al. [28]	1	Spain	Cs + MMF + PS	5 months	Fever; weight loss; peripheral oedema; splenomegaly	Anaemia	NR	Serology (IFA); BM microscopy	MA (discontinued due to intolerance), ketoconazole + allopurinol	NR
2000	Llorente et al. [29]	1	Spain	Cs + AZA + PS	48 months	Fever; splenomegaly; hepatomegaly	Pancytopenia	NR	BM microscopy	MA (discontinued due to intolerance), ketoconazole + allopurinol	NR
2001	Rodríguez-Wilhelmi et al. [30]	1	Spain	Cs + AZA + PS	60 months	Abdominal pain	Pancytopenia; hypergammaglobulinemia; elevated sCreat	NR	BM microscopy; BM culture; serology (NR)	L-AmB	Relapse
2002	Sabbatini et al. [31]	1	Italy	Cs + AZA + PS	4 months	Fever; splenomegaly; chills; malaise	Pancytopenia; hypogammaglobulinemia; elevated ESR and CRP	NR	BM microscopy	MA	Relapse
2003	Sipsas et al. [32]	1	Greece	AZA + MPSL + Cs	5 months	Fever; splenomegaly; hepatomegaly; fatigue; weight loss	Pancytopenia; hypergammaglobulinemia; elevated CRP	NR	BM microscopy, serology (IFA)	MA	NR
2005	Basset et al. [33]	8	France	Cs + AZA + PSL (*n = *2); AZA (*n = *2); Cs (*n = *2); Cs + AZA (*n = *1); AZA + PSL (*n = *1)	8 months (*n = *1); 16 months (*n = *1); 21 months (*n = *1); 24 months (*n = *1); 41 months (*n = *1); 80 months (*n = *1); 93 months (*n = *1); 144 months (*n = *1)	Fever (*n = *6); splenomegaly (*n = *2); hepatomegaly (*n = *1); weight loss (*n = *5)	Pancytopenia (*n = *4); anaemia only (*n = *2); leukopenia and thrombocytopenia only (*n = *1)	NR	BM microscopy (*n = *6); peripheral blood microscopy (*n = *1); mucosal lesion biopsy (*n = *1); PCR (*n = *4); serology (NR) (*n = *8)	AmB (*n = *2); SbV (*n = *2); L-AmB (*n = *2); SbV + L-AmB (*n = *1); untreated (*n = *1)	Relapse (*n = *1); died prior to treatment (*n = *1) or due to toxic hepatitis after SbV treatment (*n = *1)
2010	Veroux et al. [34]	5	Italy	PSL + TAC + MMF (*n = *3); TAC + SL + PSL (*n = *1); SL + MMF + PSL (*n = *1)	1 week (*n = *2); 3 weeks (*n = *2); 1 month (*n = *1)	Fever (*n = *5)	Pancytopenia (*n = *5); elevated sCreat (*n = *5)	Complete occlusion of renal vasculature; Giemsa stain positive for amastigotes	Serology (rk39 ICT) (*n = *5); graft tissue microscopy (*n = *1)	L-AmB (*n = *5)	Graft nephrectomy (*n = *1); Relapse (*n = *1)
2010	Dettwiler et al. [35]	1	Switzerland (travel to Spain)	TAC + MMF + PS	69 months	Fever; splenomegaly; hepatomegaly; anorexia; weight loss; asthenia	Pancytopenia; elevated liver enzymes, CRP and sCreat; proteinuria; microhaematuria	Numerous parasites detected within renal macrophages, confirmed on electron microscopy	BM microscopy and PCR; serology (IFA)	L-AmB	Relapse
2011	Simon et al. [36]	2	Italy	Cs + AZA + PS (*n = *1); AZA + PS (*n = *2)	118 months (*n = *1); 84 months (*n = *1)	NR	NR	NR	BM microscopy (*n = *2)	L-AmB, then MA (*n = *1); L-AmB (*n = *1)	Relapse (*n = *2)
2011	Postorino et al. [37]	1	Italy	PSL + TAC + MMF	130 months	Fever; splenomegaly; hepatomegaly	Pancytopenia; elevated CRP	NR	BM microscopy and PCR	L-AmB	NR
2013	Yücel et al [38]	1	Turkey	SL + MMF + PSL	84 months	Fever; splenomegaly; abdominal pain; cough; dysphagia; multiple skin lesions	Pancytopenia; elevated liver enzymes, CRP, sCreat and urea	NR	Nasopharyngeal biopsy microscopy; BM microscopy; sputum PCR	L-AmB	Died after treatment due to bacterial pneumonia
2014	Duvignaud et al. [39]	1	France	TAC + MMF + PS	6 months	Fever; asthenia; diarrhoea	Pancytopenia	No parasites observed	BM microscopy and PCR; serology (IFA)	L-AmB (discontinued due to intolerance), pentamidine	Relapse
2014	Pedroso et al. [40]	1	Italy	TAC + MMF + PS	216 months	Fever; chills; malaise	Pancytopenia	NR	BM microscopy	L-AmB	Relapse
2017	Pérez-Jacoiste Asín et al. [41]	5	Spain	TAC + MMF + PS (*n = *4); TAC + PS (*n = *1)	2 months (*n = *1); 10 months (*n = *1); 17 months (*n = *1); 21 months (*n = *1); 51 months (*n = *1)	Fever (*n = *5); splenomegaly (*n = *3)	Pancytopenia (*n = *5)	Amastigotes in renal tissue (*n = *??)	BM microscopy (*n = *5); serology (ELISA, rk39 ICT) (*n = *3); urinary antigen test (*n = *4)	L-AmB (*n = *5); miltefosine (*n = *1)	Relapse (*n = *4); graft failure within 1 year (*n = *1)
2017	Clavijo Sánchez et al. [42]	2	Spain	TAC + MMF + PS (*n = *2)	192 months (*n = *1); 24 months (*n = *1)	Fever (*n = *2)	Pancytopenia (*n = *1); NR (*n = *1)	NR	Lymph node biopsy microscopy (*n = *1); peripheral blood PCR (*n = *2)	L-AmB (*n = *2)	Relapse (*n = *1)
2020	Marques et al. [43]	1	Portugal	TAC + MMF + PSL	108 months	Fever; hepatomegaly; anorexia; asthenia; weight loss; nasal lesions	Pancytopenia; elevated ferritin	NR	BM microscopy and PCR; serology (IFA, ELISA); nasal biopsy microscopy and PCR	L-AmB	Died from bacteraemia during treatment
2022	Present case	1	Italy	TAC + MMF + PS	18 months	Fever; night sweat; splenomegaly	Pancytopenia; elevated CRP, sCreat and ferritin	Interstitial fibrosis and tubular atrophy; PCR positive for *Leishmania* DNA	Peripheral blood PCR; graft biopsy PCR; serology (rk39 ICR, ELISA and IFA)	L-AmB	No relapse at 48 months

*AmB* amphotericin B; *AZA* azathioprine; *BM* bone marrow; *CRP* C-reactive protein; *Cs* cyclosporin A; *ELISA* enzyme-linked immunosorbent assay; *ESR* erythrocyte sedimentation rate; *ICT* immunochromatographic test; *IFA* immunofluorescence assay; *IM* intramuscular; *L‐AmB* liposomal amphotericin B; *MA* meglumine antimoniate; *MMF* mycophenolate mofetil; *MPSL* methylprednisolone; *NR* not reported; *PSL* prednisolone; *PS* prednisone; *SbV* pentavalent antimonials; *sCreat* serum creatinine; *SL* sirolimus; *SSG* sodium stibogluconate; *TAC* tacrolimus; *Tx* transplantation; *VL* visceral leishmaniasis; *WB* Western blot

In the examined case, reactivation of a latent infection was strongly suggested by the retrospective serological analysis of the patient’s and the donor’s sera, which were collected before transplantation; this may be of importance since we recently observed the presence of a latent *Leishmania* infection in 16% of ESRD patients in haemodialysis treatment [44]. These findings call for further evaluation of the clinical utility of pre-transplant screening strategies, which are currently not recommended for this parasitic infection [4].

### VL onset and diagnosis

Development of VL is considered a late event post-transplantation, with an average VL onset at 18 months after transplants [6]. Accordingly, in southern Europe, 45 (75%) KT recipients who developed VL did so at least 6 months after the transplant (Table 1): this includes the examined case, who progressed into VL at around 18 months after receiving the graft.

Timely diagnosis of VL is critical in SOT recipients, but leishmaniasis is often overlooked in differential diagnosis, leading to treatment delay. Antinori et al. [6] reported a mean time to diagnosis of 30 days from the onset of symptoms, with peaks of up to 5 months. Time to diagnosis is seldom reported in studies of VL in KT, with only 13 (21%) case reports from southern Europe stating it (data not shown). In line with previous studies [6, 45], the current VL case was diagnosed 8 days after hospital admission, corresponding to 28 days after the symptoms’ onset.

In immunocompromised patients, diagnostic tools for VL exhibit variable performances, with serological tests showing lower sensitivity than in immunocompetent individuals and non-invasive direct diagnosis, such as molecular testing on peripheral blood, being of great value [46–49]. Nevertheless, microscopy on bone marrow samples has been the most used diagnostic method (53 cases, 87%), with PCR in peripheral or bone marrow blood carried out in 12 (20%) VL cases in KT recipients described in southern Europe, including the current case (Table 1).

### Renal involvement in VL

Renal involvement is frequent in human leishmaniasis and it is associated with increased mortality. Clinical features are diverse, mainly represented by urinary abnormalities (proteinuria, haematuria, and pyuria) and acute kidney injury (AKI), which is reported in 4–46% of VL cases. Tubular and glomerular dysfunction have also been reported, although less frequently [5, 50–53].

Renal injury can be the consequence of direct kidney involvement, but can also be caused or exacerbated by concomitant events.

Direct *Leishmania*-induced renal damage mainly results from immunological phenomena, such as the deposition of immune complexes, activation of T cells, up-regulation of adhesion molecules, inflammatory processes, but can also be caused by parasite proliferation in the kidney tissue [54, 55] Beside VL, the other causes that can contribute to the development of renal damage in VL patients are drug toxicity, presence of associated infections and haemodynamic abnormalities [51, 53, 56]. Histological examination of graft biopsies in KT recipients with VL is seldom described (Table 1); findings range from the absence of parasites [39] to diffuse interstitial inflammation and abundant parasites [35] to complete occlusion of renal vasculature [34]. The examined case shows interstitial fibrosis, tubular atrophy and glomerulosclerosis (seven out of 13 glomeruli) at histology; these chronic lesions may be related to the prolonged damage caused by the parasitic infection, considering the long-time interval between VL onset and histological evaluation. The presence of leishmanial DNA in the kidney tissue sustains this hypothesis, even though no amastigotes were detected in the lesions.

### VL treatment and monitoring

Treatment of VL in SOT recipients is mostly based on case reports or small case series. A high dose L-Amb (total dose of 40 mg/kg) is considered the therapy of choice in immunocompromised patients with VL; doses of immunosuppressive drugs should be decreased during VL treatment whenever possible [1]. In the examined case, immunosuppressants were decreased and the response to specific anti-leishmanial therapy was rapid.

qPCR on peripheral blood is considered the most useful technique for monitoring the efficacy of VL treatment and for identifying relapses in immunocompromised patients [1, 46]. In the current case, the employment of qPCR for the patient’s follow-up allowed the observation of an effective response to anti-parasitic therapy with a rapid drop of parasitaemia, with no *Leishmania* DNA detected in peripheral blood samples for the entire monitoring period (48 months) (Fig. 1a–c and data not shown). Importantly, leishmanial DNA can be detected in immunocompromised patients after treatment without clinical significance, thus caution should be taken to identify VL relapse merely on a positive PCR test [46]. qPCR should be used, with raised levels of parasitic DNA predicting relapses. It is also important to underline that qPCR results should be combined with clinical and laboratory signs to evaluate whether the patient undergo a clinically evident relapse and requires VL treatment.

## Conclusions

VL must be suspected among the opportunistic infections that can develop in SOT patients who reside in (or travelled to) *Leishmania*-endemic countries, including southern Europe. As VL exhibits high mortality in KT patients, prompt VL diagnosis and early anti-parasitic treatment are essential. PCR in peripheral blood appears to be effective for VL identification and follow-up; nevertheless, standardization and validation of a consensus protocol for molecular diagnosis and parasite load estimation is still lacking as well a consensus for screening strategies for SOT candidates in endemic regions.
